# Supplementary material for: Financial hardship among patients suffering from neglected tropical diseases: A systematic review and meta-analysis of global literature
Source: PLoS Negl Trop Dis. 2024 May 13;18(5):e0012086. doi: 10.1371/journal.pntd.0012086 (PMC11090293; doi:10.1371/journal.pntd.0012086)
Supplement: S1 Table — (DOCX) [file pntd.0012086.s002.docx]

**S1 Table. Differences from original review protocol**

| **Original review protocol** | **Difference** | **Rationale** |
| --- | --- | --- |
| - | We performed meta-analyses to calculate the pooled proportions of catastrophic health expenditure, which were measured using the same definition, e.g., direct out-of-pocket costs exceeded 10% of annual household income. | Meta-analyses were performed a posteriori after inspecting the extracted data. |
