# Supplementary material for: Financial hardship among patients suffering from neglected tropical diseases: A systematic review and meta-analysis of global literature
Source: PLoS Negl Trop Dis. 2024 May 13;18(5):e0012086. doi: 10.1371/journal.pntd.0012086 (PMC11090293; doi:10.1371/journal.pntd.0012086)
Supplement: S2 Table — (DOCX) [file pntd.0012086.s003.docx]

**S2 Table. Full search strategy**

| **Date** | **Database** | **Search term** | **Results** |
| --- | --- | --- | --- |
| 1-Jan-2023 | PubMed | ("Neglected tropical disease"[Title/Abstract] OR "Neglected tropical diseases"[Title/Abstract] OR "Neglected disease"[Title/Abstract] OR "Neglected infectious disease"[Title/Abstract] OR "Buruli ulcer"[Title/Abstract] OR "Mycobacterium ulcerans"[Title/Abstract] OR Chagas[Title/Abstract] OR "Trypanosoma cruzi"[Title/Abstract] OR "American trypanosomiasis"[Title/Abstract] OR Dengue[Title/Abstract] OR Chikungunya[Title/Abstract] OR Dracunculiasis[Title/Abstract] OR "Guinea-worm disease"[Title/Abstract] OR Echinococcosis[Title/Abstract] OR ((foodborne[Title/Abstract] OR "food borne"[Title/Abstract]) AND trematod*[Title/Abstract]) OR "Human African trypanosomiasis"[Title/Abstract] OR "Sleeping sickness"[Title/Abstract] OR "Trypanosoma brucei gambiense"[Title/Abstract] OR Leishmanias*[Title/Abstract] OR Kala-azar[Title/Abstract] OR Leprosy[Title/Abstract] OR "Hansen’s disease"[Title/Abstract] OR "Mycobacterium leprae" OR "lymphatic filariasis"[Title/Abstract] OR Elephantiasis[Title/Abstract] OR Mycetoma[Title/Abstract] OR Chromoblastomycosis[Title/Abstract] OR "Deep mycoses"[Title/Abstract] OR "Fonsecaea pedrosoi"[Title/Abstract] OR "Cladophialophora carrionii"[Title/Abstract] OR "Phialophora verrucosa"[Title/Abstract] OR Onchocerciasis[Title/Abstract] OR "River blindness"[Title/Abstract] OR "Onchocerca volvulus"[Title/Abstract] OR Rabies[Title/Abstract] OR Scabies[Title/Abstract] OR Ectoparasitos*[Title/Abstract] OR "Sarcoptes scabiei"[Title/Abstract] OR Schistosomiasis[Title/Abstract] OR "Snail fever"[Title/Abstract] OR Bilharziasis[Title/Abstract] OR ("Soil-transmitted"[Title/Abstract] AND Helminth*[Title/Abstract]) OR Ascariasis[Title/Abstract] OR Hookworm[Title/Abstract] OR Roundworm[Title/Abstract] OR Trichuriasis[Title/Abstract] OR Whipworm[Title/Abstract] OR Snake[Title/Abstract] OR Snakebite[Title/Abstract] OR Snakebite envenoming[Title/Abstract] OR Taeniasis[Title/Abstract] OR Teniasis[Title/Abstract] OR Cysticercosis[Title/Abstract] OR "Taenia solium"[Title/Abstract] OR "Taenia saginata"[Title/Abstract] OR "Taenia asiatica"[Title/Abstract] OR Trachoma[Title/Abstract] OR "Chlamydia trachomatis"[Title/Abstract] OR Yaws[Title/Abstract] OR (Endemic[Title/Abstract] AND treponematos*[Title/Abstract])) AND (catastroph* OR impoverish* OR coping OR economic consequence* OR out-of-pocket OR "out of pocket" OR ((household[Title/Abstract] OR family[Title/Abstract] OR patient[Title/Abstract]) AND (cost*[Title/Abstract] OR spending[Title/Abstract] OR expen*[Title/Abstract]))) NOT Review[Publication type] | 1566 |
| 1-Jan-2023 | Embase | ('neglected tropical disease':ti,ab OR 'neglected tropical diseases':ti,ab OR 'neglected disease':ti,ab OR 'neglected infectious disease':ti,ab OR 'buruli ulcer':ti,ab OR 'mycobacterium ulcerans':ti,ab OR chagas:ti,ab OR 'trypanosoma cruzi':ti,ab OR 'american trypanosomiasis':ti,ab OR dengue:ti,ab OR chikungunya:ti,ab OR dracunculiasis:ti,ab OR 'guinea-worm disease':ti,ab OR echinococcosis:ti,ab OR ((foodborne:ti,ab OR 'food borne':ti,ab) AND trematod*:ti,ab) OR 'african trypanosomiasis':ti,ab OR 'trypanosoma brucei gambiense':ti,ab OR leishmaniasis:ti,ab OR Kala-azar:ti,ab OR leprosy:ti,ab OR 'lymphatic filariasis':ti,ab OR elephantiasis:ti,ab OR mycetoma:ti,ab OR chromomycosis:ti,ab OR mycosis:ti,ab OR 'fonsecaea pedrosoi':ti,ab OR 'cladophialophora carrionii':ti,ab OR 'phialophora verrucosa':ti,ab OR onchocerciasis:ti,ab OR 'river blindness':ti,ab OR rabies:ti,ab OR scabies:ti,ab OR ectoparasitosis:ti,ab OR 'sarcoptes scabiei':ti,ab OR schistosomiasis:ti,ab OR 'snail fever':ti,ab OR 'soil transmitted helminth':ti,ab OR ascariasis:ti,ab OR hookworm:ti,ab OR nematode:ti,ab OR trichuriasis:ti,ab OR trichuris:ti,ab OR snake:ti,ab OR snakebite:ti,ab OR 'snakebite envenoming':ti,ab OR taeniasis:ti,ab OR cysticercosis:ti,ab OR trachoma:ti,ab OR yaws:ti,ab OR treponematosis:ti,ab) AND (catastroph* OR impoverish* OR coping OR 'economic consequences' OR 'out of pocket' OR ((household:ti,ab OR family:ti,ab OR patient:ti,ab) AND (cost*:ti,ab OR spending:ti,ab OR expen*:ti,ab))) AND [embase]/lim NOT ([embase]/lim AND [medline]/lim) AND 'article'/it | 191 |
| 1-Jan-2023 | EconLit | TI ("Neglected tropical disease" OR "Neglected tropical diseases" OR "Neglected disease" OR "Neglected infectious disease" OR "Buruli ulcer" OR "Mycobacterium ulcerans" OR Chagas OR "Trypanosoma cruzi" OR "American trypanosomiasis" OR Dengue OR Chikungunya OR Dracunculiasis OR "Guinea-worm disease" OR Echinococcosis OR ((foodborne OR "food borne") AND trematod*) OR "Human African trypanosomiasis" OR "Sleeping sickness" OR "Trypanosoma brucei gambiense" OR Leishmanias* OR Kala-azar OR Leprosy OR "Hansen’s disease" OR "Mycobacterium leprae" OR "lymphatic filariasis" OR Elephantiasis OR Mycetoma OR Chromoblastomycosis OR "Deep mycoses" OR "Fonsecaea pedrosoi" OR "Cladophialophora carrionii" OR "Phialophora verrucosa" OR Onchocerciasis OR "River blindness" OR "Onchocerca volvulus" OR Rabies OR Scabies OR Ectoparasitos* OR "Sarcoptes scabiei" OR Schistosomiasis OR "Snail fever" OR Bilharziasis OR ("Soil-transmitted" AND Helminth* ) OR Ascariasis OR Hookworm OR Roundworm OR Trichuriasis OR Whipworm OR Snake OR Snakebite OR Snakebite envenoming OR Taeniasis OR Teniasis OR Cysticercosis OR "Taenia solium" OR "Taenia saginata" OR "Taenia asiatica" OR Trachoma OR "Chlamydia trachomatis" OR Yaws OR (Endemic AND treponematos*)) AND AB (catastroph* OR impoverish* OR coping OR economic consequence* OR out-of-pocket OR "out of pocket" OR ((household OR family OR patient) AND (cost* OR spending OR expen*))) | 7 |
| 1-Jan-2023 | OpenGrey | Title ("Neglected tropical disease" OR "Neglected tropical diseases" OR "Neglected disease" OR "Neglected infectious disease" OR "Buruli ulcer" OR "Mycobacterium ulcerans" OR Chagas OR "Trypanosoma cruzi" OR "American trypanosomiasis" OR Dengue OR Chikungunya OR Dracunculiasis OR "Guinea-worm disease" OR Echinococcosis OR ((foodborne OR "food borne") AND trematod*) OR "Human African trypanosomiasis" OR "Sleeping sickness" OR "Trypanosoma brucei gambiense" OR Leishmanias* OR Kala-azar OR Leprosy OR "Hansen’s disease" OR "Mycobacterium leprae" OR "lymphatic filariasis" OR Elephantiasis OR Mycetoma OR Chromoblastomycosis OR "Deep mycoses" OR "Fonsecaea pedrosoi" OR "Cladophialophora carrionii" OR "Phialophora verrucosa" OR Onchocerciasis OR "River blindness" OR "Onchocerca volvulus" OR Rabies OR Scabies OR Ectoparasitos* OR "Sarcoptes scabiei" OR Schistosomiasis OR "Snail fever" OR Bilharziasis OR ("Soil-transmitted" AND Helminth* ) OR Ascariasis OR Hookworm OR Roundworm OR Trichuriasis OR Whipworm OR Snake OR Snakebite OR Snakebite envenoming OR Taeniasis OR Teniasis OR Cysticercosis OR "Taenia solium" OR "Taenia saginata" OR "Taenia asiatica" OR Trachoma OR "Chlamydia trachomatis" OR Yaws OR (Endemic AND treponematos*)) AND (catastroph* OR impoverish* OR coping OR economic consequence* OR out-of-pocket OR "out of pocket" OR ((household OR family OR patient) AND (cost* OR spending OR expen*))) | 0 |
| 1-Jan-2023 | EBSCO Open Dissertations | TI ("Neglected tropical disease" OR "Neglected tropical diseases" OR "Neglected disease" OR "Neglected infectious disease" OR "Buruli ulcer" OR "Mycobacterium ulcerans" OR Chagas OR "Trypanosoma cruzi" OR "American trypanosomiasis" OR Dengue OR Chikungunya OR Dracunculiasis OR "Guinea-worm disease" OR Echinococcosis OR ((foodborne OR "food borne") AND trematod*) OR "Human African trypanosomiasis" OR "Sleeping sickness" OR "Trypanosoma brucei gambiense" OR Leishmanias* OR Kala-azar OR Leprosy OR "Hansen’s disease" OR "Mycobacterium leprae" OR "lymphatic filariasis" OR Elephantiasis OR Mycetoma OR Chromoblastomycosis OR "Deep mycoses" OR "Fonsecaea pedrosoi" OR "Cladophialophora carrionii" OR "Phialophora verrucosa" OR Onchocerciasis OR "River blindness" OR "Onchocerca volvulus" OR Rabies OR Scabies OR Ectoparasitos* OR "Sarcoptes scabiei" OR Schistosomiasis OR "Snail fever" OR Bilharziasis OR ("Soil-transmitted" AND Helminth* ) OR Ascariasis OR Hookworm OR Roundworm OR Trichuriasis OR Whipworm OR Snake OR Snakebite OR Snakebite envenoming OR Taeniasis OR Teniasis OR Cysticercosis OR "Taenia solium" OR "Taenia saginata" OR "Taenia asiatica" OR Trachoma OR "Chlamydia trachomatis" OR Yaws OR (Endemic AND treponematos*)) AND TI (catastroph* OR impoverish* OR coping OR economic consequence* OR out-of-pocket OR "out of pocket" OR ((household OR family OR patient) AND (cost* OR spending OR expen*))) | 3 |
|  |  | **Total** | **1767** |
