# Supplementary material for: Financial hardship among patients suffering from neglected tropical diseases: A systematic review and meta-analysis of global literature
Source: PLoS Negl Trop Dis. 2024 May 13;18(5):e0012086. doi: 10.1371/journal.pntd.0012086 (PMC11090293; doi:10.1371/journal.pntd.0012086)
Supplement: S3 Table — (DOCX) [file pntd.0012086.s004.docx]

**S3 Table. Excluded studies with reasons**

| **Reason** | **Citation** |
| --- | --- |
| Not assessing financial catastrophes (n = 65) | 1. Amoakoh HB and Aikins M. Household cost of out-patient treatment of Buruli ulcer in Ghana: a case study of Obom in Ga South Municipality. BMC Health Serv Res 2013; 13: 507. 20131205. DOI: 10.1186/1472-6963-13-507. 2. Anoopa Sharma D, Bern C, Varghese B, et al. The economic impact of visceral leishmaniasis on households in Bangladesh. Trop Med Int Health 2006; 11: 757-764. DOI: 10.1111/j.1365-3156.2006.01604.x. 3. Grietens KP, Boock AU, Peeters H, et al. "It is me who endures but my family that suffers": social isolation as a consequence of the household cost burden of Buruli ulcer free of charge hospital treatment. PLoS Negl Trop Dis 2008; 2: e321. 20081015. DOI: 10.1371/journal.pntd.0000321. 4. Harving ML and Rönsholt FF. The economic impact of dengue hemorrhagic fever on family level in Southern Vietnam. Dan Med Bull 2007; 54: 170-172. 5. Lee JS, Mogasale V, Lim JK, et al. A multi-country study of the economic burden of dengue fever: Vietnam, Thailand, and Colombia. PLoS Negl Trop Dis 2017; 11: e0006037. 20171030. DOI: 10.1371/journal.pntd.0006037. 6. Lee JS, Mogasale V, Lim JK, et al. A multi-country study of the economic burden of dengue fever based on patient-specific field surveys in Burkina Faso, Kenya, and Cambodia. PLoS Negl Trop Dis 2019; 13: e0007164. 20190228. DOI: 10.1371/journal.pntd.0007164. 7. Meheus F, Boelaert M, Baltussen R, et al. Costs of patient management of visceral leishmaniasis in Muzaffarpur, Bihar, India. Trop Med Int Health 2006; 11: 1715-1724. DOI: 10.1111/j.1365-3156.2006.01732.x. 8. Ozaki M, Islam S, Rahman KM, et al. Economic consequences of post-kala-azar dermal leishmaniasis in a rural Bangladeshi community. Am J Trop Med Hyg 2011; 85: 528-534. DOI: 10.4269/ajtmh.2011.10-0683. 9. Ramaiah KD, Guyatt H, Ramu K, et al. Treatment costs and loss of work time to individuals with chronic lymphatic filariasis in rural communities in south India. Trop Med Int Health 1999; 4: 19-25. DOI: 10.1046/j.1365-3156.1999.00351.x. 10. Rijal S, Koirala S, Van der Stuyft P, et al. The economic burden of visceral leishmaniasis for households in Nepal. Trans R Soc Trop Med Hyg 2006; 100: 838-841. 20060110. DOI: 10.1016/j.trstmh.2005.09.017. 11. Salmon-Mulanovich G, Blazes DL, Lescano AG, et al. Economic Burden of Dengue Virus Infection at the Household Level Among Residents of Puerto Maldonado, Peru. Am J Trop Med Hyg 2015; 93: 684-690. 20150727. DOI: 10.4269/ajtmh.14-0755. 12. Sarnoff R, Desai J, Desjeux P, et al. The economic impact of visceral leishmaniasis on rural households in one endemic district of Bihar, India. Trop Med Int Health 2010; 15 Suppl 2: 42-49. 20100506. DOI: 10.1111/j.1365-3156.2010.02516.x. 13. Sonali Fernando E, Headley TY, Tissera H, et al. Household and Hospitalization Costs of Pediatric Dengue Illness in Colombo, Sri Lanka. Am J Trop Med Hyg 2021; 105: 110-116. 20210517. DOI: 10.4269/ajtmh.20-1179. 14. Tembei AM, Kengne-Ouaffo JA, Ngoh EA, et al. A Comparative Analysis of Economic Cost of Podoconiosis and Leprosy on Affected Households in the Northwest Region of Cameroon. Am J Trop Med Hyg 2018; 98: 1075-1081. 20180215. DOI: 10.4269/ajtmh.17-0931. 15. Tozan Y, Ratanawong P, Sewe MO, et al. Household costs of hospitalized dengue illness in semi-rural Thailand. PLoS Negl Trop Dis 2017; 11: e0005961. 20170922. DOI: 10.1371/journal.pntd.0005961. 16. Wijerathna T, Gunathilaka N and Gunawardena K. The Economic Impact of Cutaneous Leishmaniasis in Sri Lanka. Biomed Res Int 2018; 2018: 3025185. 20181011. DOI: 10.1155/2018/3025185. 17. Xiong M, Li M, Zheng D, et al. Evaluation of the economic burden of leprosy among migrant and resident patients in Guangdong Province, China. BMC Infect Dis 2017; 17: 760. 20171211. DOI: 10.1186/s12879-017-2869-8. 18. Alvis-Zakzuk NJ, Díaz-Jiménez D, Castillo-Rodríguez L, et al. Economic Costs of Chikungunya Virus in Colombia. Value Health Reg Issues 2018; 17: 32-37. 20180406. DOI: 10.1016/j.vhri.2018.01.004. 19. Amparo ACB, Jayme SI, Roces MCR, et al. The evaluation of Animal Bite Treatment Centers in the Philippines from a patient perspective. PLoS One 2018; 13: e0200873. 20180726. DOI: 10.1371/journal.pone.0200873. 20. Babo Martins S, Bolon I, Alcoba G, et al. Assessment of the effect of snakebite on health and socioeconomic factors using a One Health perspective in the Terai region of Nepal: a cross-sectional study. Lancet Glob Health 2022; 10: e409-e415. DOI: 10.1016/s2214-109x(21)00549-0. 21. Babu BV and Nayak AN. Treatment costs and work time loss due to episodic adenolymphangitis in lymphatic filariasis patients in rural communities of Orissa, India. Trop Med Int Health 2003; 8: 1102-1109. DOI: 10.1046/j.1360-2276.2003.01146.x. 22. Babu BV, Nayak AN, Dhal K, et al. The economic loss due to treatment costs and work loss to individuals with chronic lymphatic filariasis in rural communities of Orissa, India. Acta Trop 2002; 82: 31-38. DOI: 10.1016/s0001-706x(02)00030-x. 23. Bay V, Rezapour A, Jafari M, et al. Healthcare utilization patterns and economic burden of animal bites: A cross-sectional study. Journal of Acute Disease 2021; 10: 142-146. Article. DOI: 10.4103/2221-6189.320962. 24. Bukachi SA, Wandibba S and Nyamongo IK. The socio-economic burden of human African trypanosomiasis and the coping strategies of households in the South Western Kenya foci. PLoS Negl Trop Dis 2017; 11: e0006002. 20171026. DOI: 10.1371/journal.pntd.0006002. 25. Castro Rodriguez R, Galera-Gelvez K, López Yescas JG, et al. Costs of dengue to the health system and individuals in Colombia from 2010 to 2012. Am J Trop Med Hyg 2015; 92: 709-714. 20150209. DOI: 10.4269/ajtmh.14-0386. 26. Chatterjee SS, Sharma A, Choudhury S, et al. Dengue fever in a south Asian metropolis: a report on 219 cases. Iran J Microbiol 2017; 9: 174-185. 27. Clark DV, Mammen MP, Jr., Nisalak A, et al. Economic impact of dengue fever/dengue hemorrhagic fever in Thailand at the family and population levels. Am J Trop Med Hyg 2005; 72: 786-791. 28. Eneanya OA, Garske T and Donnelly CA. The social, physical and economic impact of lymphedema and hydrocele: a matched cross-sectional study in rural Nigeria. BMC Infect Dis 2019; 19: 332. 20190423. DOI: 10.1186/s12879-019-3959-6. 29. H NR, George R, Eapen EP, et al. A comparison of economic aspects of hospitalization versus ambulatory care in the management of neuritis occurring in lepra reaction. Int J Lepr Other Mycobact Dis 2004; 72: 448-456. DOI: 10.1489/1544-581x(2004)72<448:Acoeao>2.0.Co;2. 30. Haradanhalli RS, AN DH and Varadappa ST. Cost of rabies post exposure prophylaxis in different healthcare settings in six states of India. Indian J Public Health 2019; 63: S44-s47. DOI: 10.4103/ijph.IJPH_366_19. 31. Hasan SM, Basher A, Molla AA, et al. The impact of snake bite on household economy in Bangladesh. Trop Doct 2012; 42: 41-43. 20120105. DOI: 10.1258/td.2011.110137. 32. Hounsome N, Hassan R, Bakhiet SM, et al. Role of socioeconomic factors in developing mycetoma: Results from a household survey in Sennar State, Sudan. PLoS Negl Trop Dis 2022; 16: e0010817. 20221017. DOI: 10.1371/journal.pntd.0010817. 33. Huy R, Wichmann O, Beatty M, et al. Cost of dengue and other febrile illnesses to households in rural Cambodia: a prospective community-based case-control study. BMC Public Health 2009; 9: 155. 20090527. DOI: 10.1186/1471-2458-9-155. 34. Ibe O, Onwujekwe O, Uzochukwu B, et al. Exploring Consumer Perceptions and Economic Burden of Onchocerciasis on Households in Enugu State, South-East Nigeria. PLoS Negl Trop Dis 2015; 9: e0004231. 20151130. DOI: 10.1371/journal.pntd.0004231. 35. Kasturiratne A, Pathmeswaran A, Wickremasinghe AR, et al. The socio-economic burden of snakebite in Sri Lanka. PLoS Negl Trop Dis 2017; 11: e0005647. 20170706. DOI: 10.1371/journal.pntd.0005647. 36. Kaur J, Yadav CP, Chauhan NM, et al. Economic burden estimation associated with dengue and chikungunya in Gujarat, India. J Family Med Prim Care 2022; 11: 5393-5403. 20221014. DOI: 10.4103/jfmpc.jfmpc_694_21. 37. Khun S and Manderson L. Poverty, user fees and ability to pay for health care for children with suspected dengue in rural Cambodia. Int J Equity Health 2008; 7: 10. 20080425. DOI: 10.1186/1475-9276-7-10. 38. Kopparty SN, Kurup AM and Sivaram M. Problems and coping strategies of families having patients with and without deformities. Indian J Lepr 1995; 67: 133-152. 39. Krishnamoorthy K. Estimated costs of acute adenolymphangitis to patients with chronic manifestations of bancroftian filariasis in India. Indian J Public Health 1999; 43: 58-63. 40. Kusuma YS and Babu BV. The costs of seeking healthcare: Illness, treatment seeking and out of pocket expenditures among the urban poor in Delhi, India. Health Soc Care Community 2019; 27: 1401-1420. 20190625. DOI: 10.1111/hsc.12792. 41. Legorreta-Soberanis J, Paredes-Solís S, Morales-Pérez A, et al. Household costs of dengue illness: secondary outcomes from a randomised controlled trial of dengue prevention in Guerrero state, Mexico. BMC Public Health 2017; 17: 411. 20170530. DOI: 10.1186/s12889-017-4304-x. 42. Magalhães SFV, Peixoto HM, de Almeida Gonçalves Sachett J, et al. Snakebite envenomation in the Brazilian Amazon: a cost-of-illness study. Trans R Soc Trop Med Hyg 2020; 114: 635-642. DOI: 10.1093/trstmh/traa005. 43. Martelli CM, Siqueira JB, Jr., Parente MP, et al. Economic Impact of Dengue: Multicenter Study across Four Brazilian Regions. PLoS Negl Trop Dis 2015; 9: e0004042. 20150924. DOI: 10.1371/journal.pntd.0004042. 44. Mia MS, Begum RA, Er AC, et al. ASSESSING THE COST BURDEN OF DENGUE INFECTION TO HOUSEHOLDS IN SEREMBAN, MALAYSIA. Southeast Asian J Trop Med Public Health 2016; 47: 1167-1176. 45. Mwiinde AM, Simuunza M, Namangala B, et al. Estimating the economic and social consequences for patients diagnosed with human African trypanosomiasis in Muchinga, Lusaka and Eastern Provinces of Zambia (2004-2014). Infect Dis Poverty 2017; 6: 150. 20171010. DOI: 10.1186/s40249-017-0363-6. 46. Nadjib M, Setiawan E, Putri S, et al. Economic burden of dengue in Indonesia. PLoS Negl Trop Dis 2019; 13: e0007038. 20190110. DOI: 10.1371/journal.pntd.0007038. 47. Nanda B and Krishnamoorthy K. Treatment seeking behaviour and costs due to acute and chronic forms of lymphatic filariasis in urban areas in south India. Trop Med Int Health 2003; 8: 56-59. DOI: 10.1046/j.1365-3156.2003.00962.x. 48. Nandha B and Krishnamoorthy K. Cost of illness due to Chikungunya during 2006 outbreak in a rural area in Tamil Nadu. Indian J Public Health 2009; 53: 209-213. 49. Nhi TNY and Trung VQ. The economic value of informal care for dengue patients in vietnam. International Journal of Research in Ayurveda and Pharmacy 2016; 7: 101-106. Article. DOI: 10.7897/2277-4343.076249. 50. Nujum ZT, Beegum MS, Meenakshy V, et al. Cost analysis of dengue from a State in south India. Indian J Med Res 2020; 152: 490-497. DOI: 10.4103/ijmr.IJMR_1641_18. 51. Okanurak K, Sornmani S and Indaratna K. The cost of dengue hemorrhagic fever in Thailand. Southeast Asian J Trop Med Public Health 1997; 28: 711-717. 52. Ooms GI, van Oirschot J, Waldmann B, et al. The Burden of Snakebite in Rural Communities in Kenya: A Household Survey. Am J Trop Med Hyg 2021; 105: 828-836. 20210719. DOI: 10.4269/ajtmh.21-0266. 53. Rafique I, Nadeem Saqib MA, Munir MA, et al. Economic burden of dengue in four major cities of Pakistan during 2011. J Pak Med Assoc 2015; 65: 256-259. 54. Salimi M, Saghafipour A, Parsa HH, et al. Economic burden evaluation of cutaneous leishmaniasis in iran. Shiraz E Medical Journal 2019; 20. Article. DOI: 10.5812/semj.82810. 55. Sawers L, Stillwaggon E, Chiphwanya J, et al. Economic benefits and costs of surgery for filarial hydrocele in Malawi. PLoS Negl Trop Dis 2020; 14: e0008003. 20200325. DOI: 10.1371/journal.pntd.0008003. 56. Sharma SK, Chappuis F, Jha N, et al. Impact of snake bites and determinants of fatal outcomes in southeastern Nepal. Am J Trop Med Hyg 2004; 71: 234-238. 57. Shwiff SA, Sterner RT, Jay MT, et al. Direct and indirect costs of rabies exposure: a retrospective study in southern California (1998-2002). J Wildl Dis 2007; 43: 251-257. DOI: 10.7589/0090-3558-43.2.251. 58. Sundar S, Arora R, Singh SP, et al. Household cost-of-illness of visceral leishmaniasis in Bihar, India. Trop Med Int Health 2010; 15 Suppl 2: 50-54. 20100506. DOI: 10.1111/j.1365-3156.2010.02520.x. 59. Tam PT, Dat NT, Huu le M, et al. High household economic burden caused by hospitalization of patients with severe dengue fever cases in Can Tho province, Vietnam. Am J Trop Med Hyg 2012; 87: 554-558. 20120723. DOI: 10.4269/ajtmh.2012.12-0101. 60. Tekola F, Mariam DH and Davey G. Economic costs of endemic non-filarial elephantiasis in Wolaita Zone, Ethiopia. Trop Med Int Health 2006; 11: 1136-1144. DOI: 10.1111/j.1365-3156.2006.01658.x. 61. Tozan Y, Headley TY, Sewe MO, et al. A Prospective Study on the Impact and Out-of-Pocket Costs of Dengue Illness in International Travelers. Am J Trop Med Hyg 2019; 100: 1525-1533. DOI: 10.4269/ajtmh.18-0780. 62. Vaiyapuri S, Vaiyapuri R, Ashokan R, et al. Snakebite and its socio-economic impact on the rural population of Tamil Nadu, India. PLoS One 2013; 8: e80090. 20131121. DOI: 10.1371/journal.pone.0080090. 63. Van Damme W, Van Leemput L, Por I, et al. Out-of-pocket health expenditure and debt in poor households: evidence from Cambodia. Trop Med Int Health 2004; 9: 273-280. DOI: 10.1046/j.1365-3156.2003.01194.x. 64. Xu M, Chang N, Tu T, et al. Economic burden of dengue fever in China: A retrospective research study. PLoS Negl Trop Dis 2022; 16: e0010360. 20220520. DOI: 10.1371/journal.pntd.0010360. 65. Zubieta-Zavala A, López-Cervantes M, Salinas-Escudero G, et al. Economic impact of dengue in Mexico considering reported cases for 2012 to 2016. PLoS Negl Trop Dis 2018; 12: e0006938. 20181214. DOI: 10.1371/journal.pntd.0006938. |
| Not using primary data collection (n = 6) | 1. Singer BH, Ryff CD. Neglected tropical diseases, neglected data sources, and neglected issues. PLoS Negl Trop Dis. 2007;1(2):e104. 2. Onuh W, Cabanacan-Salibay C, Manaig P. Economic costs and burden of dengue disease in cavite province, Philippines. Scientia Medica. 2016;26(2):1-11. 3. Akbar NA, Assiri AM, Shabouni OI, Alwafi OM, Al-Raddadi R, M HA, et al. The economic burden of dengue fever in the Kingdom of Saudi Arabia. PLoS Negl Trop Dis. 2020;14(11):e0008847. 4. Kajimoto Y, Kitajima T. Patient and National Economic Burden of Dengue in Japan: Results from Japanese National Claims Database. Am J Trop Med Hyg. 2020;102(6):1237-43. 5. Koporc KM, Hotchkiss DR, Stoecker CF, McFarland DA, Carton T. Assessing the effects of disease-specific programs on health systems: An analysis of the Bangladesh Lymphatic Filariasis Elimination Program's impacts on health service coverage and catastrophic health expenditure. PLoS Negl Trop Dis. 2021;15(11):e0009894. 6. Sambo M, Cleaveland S, Ferguson H, Lembo T, Simon C, Urassa H, et al. The burden of rabies in Tanzania and its impact on local communities. PLoS Negl Trop Dis. 2013;7(11):e2510. |
| Not assessing patients’ and household costs (n = 3) | 1. Chippaux JP, Banzou A, Agbede K. [Social and economic impact of dracunculosis: a longitudinal study carried out in 2 villages in Benin]. Bull World Health Organ. 1992;70(1):73-8. 2. Panmei K, Joseph AK, Rose W, Abraham OC, Mathuram AJ, Kumar S, et al. Direct cost of illness for dengue in hospitalized children and adults at a referral hospital in India. Int J Infect Dis. 2019;84s:S64-s7. 3. Basinger SC, Khan A, Ahmed H, Afzal MS, Simsek S, Budke CM. Estimation of the monetary burden of treated human cystic echinococcosis in Pakistan. Acta Trop. 2021;222:106026. |
| Not original articles (n = 2) | 1. Olivera MJ, Chaverra KA. New Diagnostic Algorithm for Chagas Disease: Impact on Access to Diagnosis and Out-of-Pocket Expenditures in Colombia. Iran J Public Health. 2019;48(7):1379-81. 2. Deepak S, Gopal PK, Hisch E. Consequences of leprosy and socio-economic rehabilitation. Lepr Rev. 2000;71(4):417-9. |
