# Supplementary material for: Financial hardship among patients suffering from neglected tropical diseases: A systematic review and meta-analysis of global literature
Source: PLoS Negl Trop Dis. 2024 May 13;18(5):e0012086. doi: 10.1371/journal.pntd.0012086 (PMC11090293; doi:10.1371/journal.pntd.0012086)
Supplement: S4 Table — (DOCX) [file pntd.0012086.s005.docx]

**S4 Table. Quality assessment using Larg, A., and Moss, J. R. (2011) Cost-of-illness studies: a guide to critical evaluation.**

| **Items** | **Chukwu,  2017 [20]** | **Gopalan,  2009 [21]** | **Vijayakumar,  2013 [26]** | **McBride,  2019 [22]** | **Adhikari,  2009 [8]** | **Meheus,  2013 [15]** | **Uranw,  2013 [25]** | **Chandler,  2015 [19]** | **Tiwari,  2018 [23]** | **Tripathy,  2020 [24]** |
| --- | --- | --- | --- | --- | --- | --- | --- | --- | --- | --- |
| **(1) Analytical framework: what costs should have been measured?** |  |  |  |  |  |  |  |  |  |  |
| (a) What was the motivation and perspective of the study? | **Yes** | **Yes** | **Yes** | **Yes** | **Yes** | **Yes** | **Yes** | **Yes** | **Yes** | **Yes** |
| (b) Was the appropriate epidemiologic approach taken? | **Yes** | **Yes** | **Yes** | **Yes** | **Yes** | **Yes** | **Yes** | **Yes** | **Yes** | **Yes** |
| (c) Was the study question well specified? | **Partly  yes** | **Partly  Yes** | **Partly  Yes** | **Yes** | **Yes** | **Yes** | **Yes** | **Yes** | **Yes** | **Yes** |
| **(2) Methodology and data: how well were resource use and productivity losses measured?** | | |  |  |  |  |  |  |  |  |
| (a) Was an appropriate method(s) of quantification used, such that | **Partly  Yes** | **Yes** | **Yes** | **Yes** | **Yes** | **Yes** | **Yes** | **Yes** | **Yes** | **Yes** |
| (b) Was the resource quantification method(s) well executed? | **Partly  Yes** | **Yes** | **Yes** | **Yes** | **Yes** | **Yes** | **Yes** | **Yes** | **Yes** | **Yes** |
| (c) Were healthcare resources valued appropriately? | **Yes** | **Yes** | **Yes** | **Yes** | **Yes** | **Yes** | **Yes** | **Yes** | **Yes** | **Yes** |
| (d) Was the approach for valuing production losses justified, and assumptions valid? | **Not included** | **Yes** | **Yes** | **Not  included** | **Yes** | **Yes** | **Yes** | **Yes** | **Yes** | **Not  included** |
| (e) Was the inclusion of intangible costs appropriate: | **Not included** | **Not included** | **Not included** | **Not included** | **Not included** | **Not included** | **Not included** | **Not included** | **Not included** | **Not included** |
| **(3) Analysis and reporting** |  |  |  |  |  |  |  |  |  |  |
| (a) Did the analysis address the study question? | **Partly**  **Yes** | **Yes** | **Yes** | **Yes** | **Yes** | **Yes** | **Yes** | **Yes** | **Yes** | **Yes** |
| (b) Was a range of estimates presented? | **Yes** | **Yes** | **Yes** | **Yes** | **Yes** | **Yes** | **Yes** | **Yes** | **Yes** | **Yes** |
| (c) Were the main uncertainties identified? | **Yes** | **Yes** | **Yes** | **Yes** | **Yes** | **Yes** | **Yes** | **Yes** | **Yes** | **Yes** |
| (d) Was a sensitivity analysis performed on: | **Not included** | **Not included** | **Not included** | **Not included** | **Not included** | **Not included** | **Not included** | **Not included** | **Not included** | **Not included** |
| (e) Was adequate documentation and justification given for cost components, data and sources, assumptions and methods? | **Yes** | **Yes** | **Yes** | **Yes** | **Yes** | **Yes** | **Yes** | **Yes** | **Yes** | **Yes** |
| (f) Was uncertainty around the estimates and its implications adequately discussed? | **Yes** | **Yes** | **Yes** | **Yes** | **Yes** | **Yes** | **Yes** | **Yes** | **Yes** | **Yes** |
| (g) Were important limitations discussed regarding the cost components, data, assumptions and methods? | **Yes** | **Yes** | **Yes** | **Yes** | **Yes** | **Yes** | **Yes** | **Yes** | **Yes** | **Yes** |
| (h) Were the results presented at the appropriate level of detail to answer the study question (cost components; disease subtypes, severity, stage; subpopulation groups, cost bearers)? | **Partly**  **Yes** | **Yes** | **Yes** | **Partly**  **Yes** | **Yes** | **Yes** | **Yes** | **Yes** | **Partly  Yes** | **Yes** |

From: Larg A, Moss JR. Cost-of-illness studies: a guide to critical evaluation. PharmacoEconomics. 2011;29(8):653-71.[13]
