# Supplementary figures and images for: Financial hardship among patients suffering from neglected tropical diseases: A systematic review and meta-analysis of global literature
Source: PLoS Negl Trop Dis. 2024 May 13;18(5):e0012086. doi: 10.1371/journal.pntd.0012086 (PMC11090293; doi:10.1371/journal.pntd.0012086)

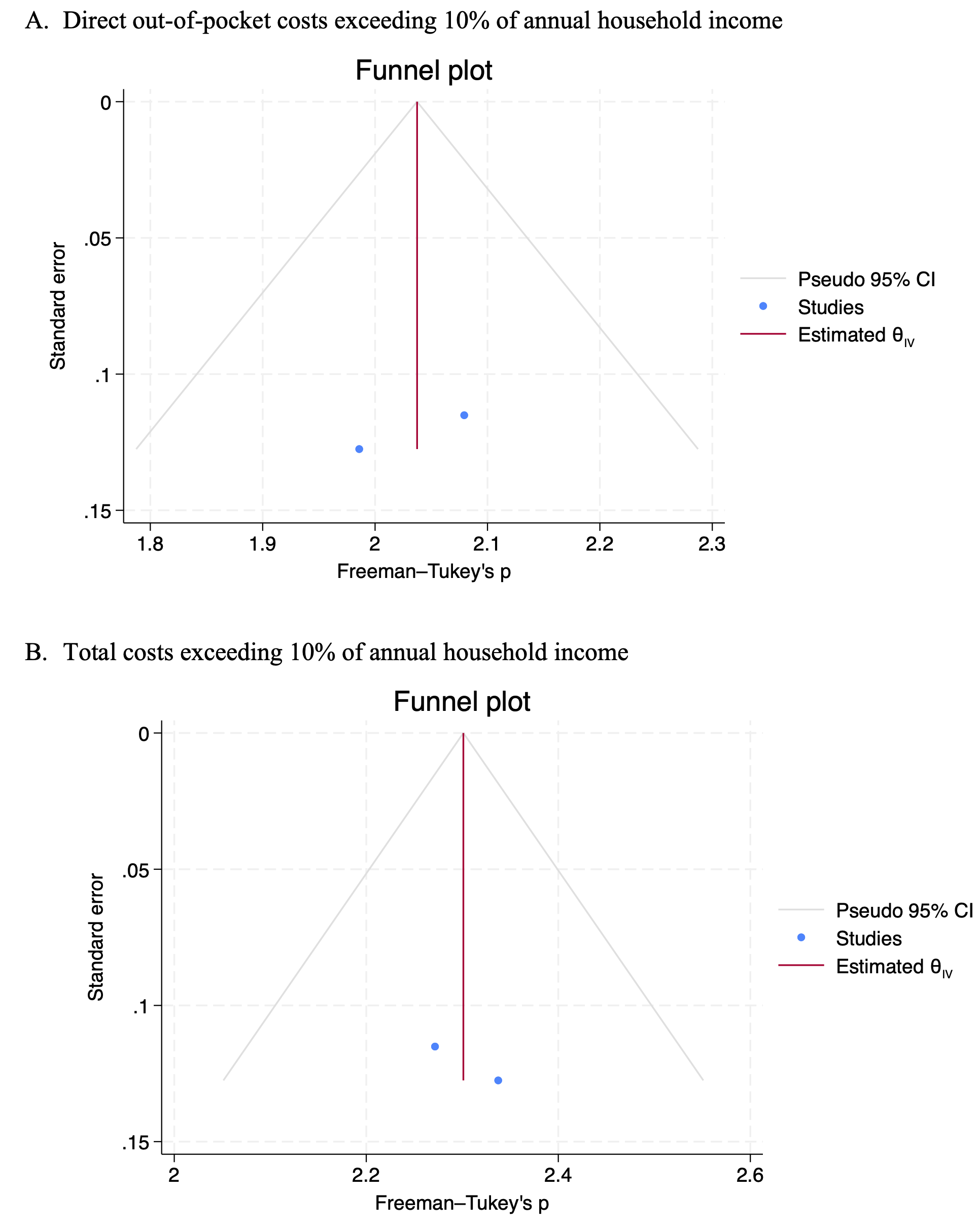

Supplement: S1 Fig — (TIFF) [file pntd.0012086.s006.tiff]

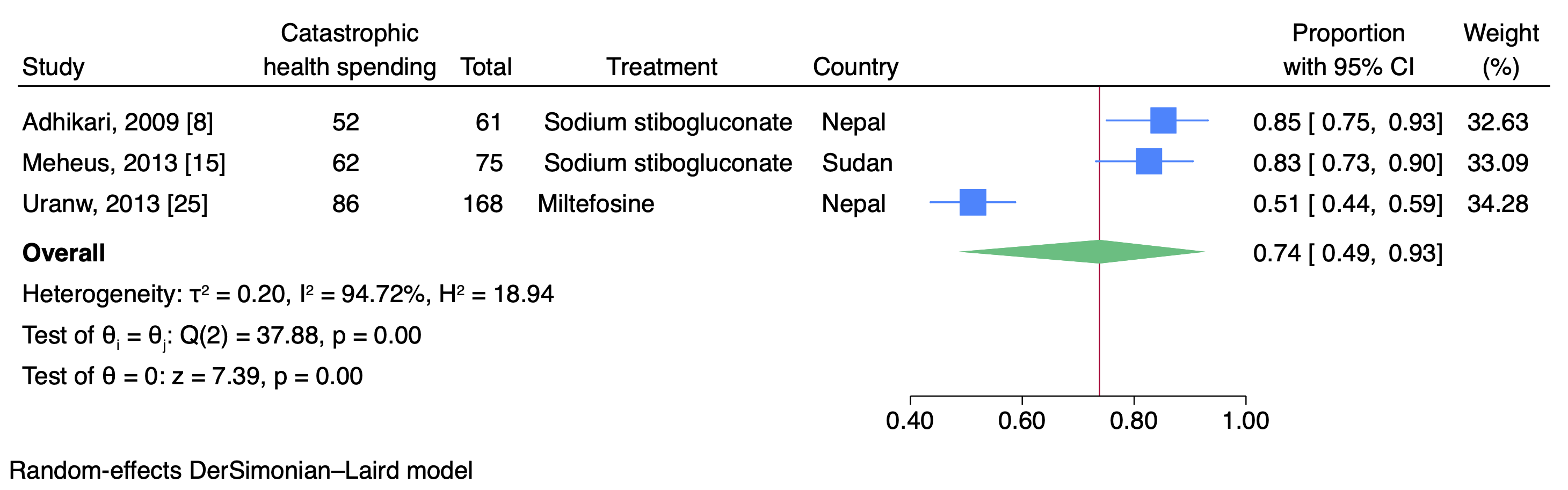

Supplement: S2 Fig — (TIFF) [file pntd.0012086.s007.tiff]
