# Supplementary material for: Financial hardship among patients suffering from neglected tropical diseases: A systematic review and meta-analysis of global literature
Source: PLoS Negl Trop Dis. 2024 May 13;18(5):e0012086. doi: 10.1371/journal.pntd.0012086 (PMC11090293; doi:10.1371/journal.pntd.0012086)
Supplement: Attachment — Submitted filename: R1_SR CHE NTDs_Author response.docx [file pntd.0012086.s008.docx]

**AUTHOR RESPONSE LETTER**

**Journal: PLOS Neglected Tropical Diseases**

**Manuscript ID: PNTD-D-23-01395.R1**

**Title: Financial Hardship among Patients Suffering from Neglected Tropical Diseases: A Systematic Review and Meta-analysis of Global Literature**

Dear Editor,

On behalf of all the authors, I would like to resubmit our revised manuscript entitled: “Financial Hardship among Patients Suffering from Neglected Tropical Diseases: A Systematic Review and Meta-analysis of Global Literature” (PNTD-D-23-01395.R1). The useful comments forwarded from the editors and reviewers are highly appreciated. We have revised our manuscript and responded point-by-point to each issue raised by the reviewers. We appreciate all valuable comments from the reviewers and have tried our best to incorporate them in this revised version.

Thank you for considering this paper for publication.

Sincerely yours,

Nathorn Chaiyakunapruk, Pharm.D., Ph.D.

Department of Pharmacotherapy, University of Utah College of Pharmacy

Email: [Nathorn.Chaiyakunapruk@utah.edu](mailto:Nathorn.Chaiyakunapruk@utah.edu)

**Response to Reviewer 1 Comments**

I read the manuscript with interest and the study poses very important research question. I have few below-mentioned suggestions for the improvement of the manuscript.

**Response**

We would like to thank you for your valuable comments and suggestions. We have revised our manuscript and responded point-by-point to each issue you raised.

**Comment 1**

In abstract, line "Meta-analysis showed that CHE risk due to VL was 73% (95% CI; 65–80%, n = 2, I^2^ = 0.00%)," I^2^ may not be clear to readers of the journal. Please specify what does I^2^ denotes.

**Response 1**

Thank you for pointing this out. We have clarified the I^2^. Please see the changes to the abstract below.

| **Original version** | **Revised version** |
| --- | --- |
| Abstract, Line 40-43  **Methods:** We searched PubMed, EMBASE, EconLit, OpenGrey, and EBSCO Open Dissertations, for articles reporting financial catastrophes from NTDs from database inception to January 1, 2023. We summarized the study findings and methodological characteristics. Meta-analyses were performed to pool the proportions of CHE. | Abstract, Line 40-44  **Methods:** We searched PubMed, EMBASE, EconLit, OpenGrey, and EBSCO Open Dissertations, for articles reporting financial hardship caused by NTDs from database inception to January 1, 2023. We summarized the study findings and methodological characteristics. Meta-analyses were performed to pool the prevalence of CHS. Heterogeneity was evaluated using the I² statistic. |

**Comment 2**

In abstract, line "Costs of VL impoverished approximately one-fifth of households."
Mention N.

**Response 2**

We have revised our abstract and author summary accordingly. Please see the changes to the abstract below.

| **Original version** | **Revised version** |
| --- | --- |
| Abstract, Line 51-52  Costs of VL impoverished approximately one-fifth of households. | Abstract, Line 52-53  Costs of visceral leishmaniasis impoverished 20-26% of the 61 households investigated, depending on the costs captured. |
| Author summary, Line 68-69  Costs of VL impoverished approximately one-fifth of households. | Author summary, Line 68-69  Costs of visceral leishmaniasis impoverished 20-26% of the 61 households investigated, depending on the costs captured. |
| Results, Line 279-281  Costs of visceral leishmaniasis impoverished approximately one-fifth of households depending on the OOP costs captured (20% medical costs, 21% medical and transportation costs, 26% direct OOP costs), as shown in Table 2. | Results, Line 309-311  Costs of visceral leishmaniasis impoverished 20-26% of the 61 households investigated, depending on the costs captured (20% medical costs, 21% medical and transportation costs, 26% direct costs), as shown in Table 2. |

**Comment 3**

Introduction needs to be elaborated more to highlight the rationale and importance of the study. The outcomes of the study include CHE, impoverishment, and coping strategies as well. However, in introduction section only CHE has been explained.

**Response 3**

We agree with your comment and have revised our introduction accordingly. Please see the changes to the manuscript below.

| **Original version** | **Revised version** |
| --- | --- |
| Introduction, Line 90-99  Financial catastrophes are usually quantified as catastrophic health expenditure (CHE). CHE can be defined as the proportion of the OOP costs exceeding a specific threshold of the total household income or non-subsistent household income.(4, 5) CHE is a well-established indicator for financial risk protection of the healthcare system, which was an essential dimension of the UHC.(6) CHE due to NTDs is vital in this respect, as most people affected by NTDs are relatively vulnerable groups who face inequitable access to healthcare. (7)Providing coverage to these groups effectively strengthens the financial risk protection of the health system, which is one of the most critical dimensions of the UHC.(6) Especially since some types of NTD are closely related to CHE, improving their financial protection may help attain UHC, especially for LMICs.(8) | Introduction, Line 89-107  Financial hardship is usually quantified as catastrophic health spending (CHS) (as known as catastrophic health expenditure) and impoverishment. CHS is as the proportion of households with OOP costs incurred by a specific disease that exceed a specific threshold of the total household income or expenditure (budget share approach) or non-subsistent household expenditure (capacity-to-pay approach). Impoverishment is when the OOP costs push households below the poverty line.(4-6) CHS and impoverishment are well-established indicators for the financial risk protection of the healthcare system, which was an essential dimension of the UHC as indicated under the SDG 3.8.2 indicators.(1,7)  Financial hardship. poses a greater challenge for individuals affected by NTDs, as they frequently reside in poverty before the onset of the disease. To evaluate the long-term economic risk imposed by health spending on NTDs, it is important to understand the coping strategies of this population. Literature has shown that coping strategies, such as seeking financial assistance through loans or selling their assets, could push households into or further into poverty if it impacts their productivity.(8) Thus, providing coverage to these groups effectively strengthens the financial risk protection of the health system.(7) Since some types of NTD are closely related to financial hardship, improving their financial protection may help attain UHC, especially for LMICs.(9) |

**Comment 4**

The manuscript contains many grammatical errors. Authors are advised to use professional editing tools to rectify all such errors.

**Response 4**

Thank you for pointing this out. Our manuscript has been proofread by a native English speaker.

**Comment 5**

Line 75-77, "In 2021, the World Health Organization (WHO) reported that 1.65 billion people required mass or individual treatment and care for neglected tropical diseases (NTDs) as they faced human, social, and economic burdens incurred by the diseases" What do the author mean by human burden incurred by the diseases?

**Response 5**

Thank you for bringing this to our attention. The term "human burden" (or humanistic burden) refers to the measurable negative effects on the quality of life and ability to fulfill the roles of patients and their caregivers. To better convey our message, we have updated the term to "humanistic burden".

| **Original version** | **Revised version** |
| --- | --- |
| Introduction, Line 75-77  In 2021, the World Health Organization (WHO) reported that 1.65 billion people required mass or individual treatment and care for neglected tropical diseases (NTDs) as they faced human, social, and economic burdens incurred by the diseases. | Introduction, Line 75-77  In 2021, the World Health Organization (WHO) reported that 1.65 billion people required treatment and care for neglected tropical diseases (NTDs) as they faced humanistic, social, and economic burdens incurred by the diseases. |

**Comment 6**

6) Line 77-78, "NTDs are defined as a diverse group of diseases whose impact on impoverished communities in (sub-) tropical areas.(1)" is incomplete and poorly framed.

**Response 6**

We have rewritten our statements. Please see the changes to the manuscript below.

| **Original version** | **Revised version** |
| --- | --- |
| Introduction Line, 77-78  NTDs are defined as a diverse group of diseases whose impact on impoverished communities in  (sub-) tropical areas.(1) | Introduction, Line 77-78  NTDs are a diverse group of diseases that mainly affect underprivileged communities in tropical and subtropical areas.(1) |

**Comment 7**

Line 80-82, "For example, as in the study reported before the first road map was published, every low-income country was affected by at least five NTDs.(2)" The line is difficult to comprehend.

**Response 7**

We have rewritten our manuscript accordingly. Please see the changes to the manuscript below.

| **Original version** | **Revised version** |
| --- | --- |
| Introduction, Line 80-82  For example, as in the study reported before the first road map was published, every low-income country was affected by at least five NTDs.(2) | Introduction, Line 80-81  It has been reported that every low-income country is affected by at least five NTDs |

**Comment 8**

Line 183-185, "We performed meta-analyses to calculate the pooled proportions of CHE, which were measured using the same definition, e.g., direct OOP costs exceeded 10% of annual household income." In literature, there is no consensus regarding the threshold at which CHE is calculated. Were there any studies that estimated CHE at any other threshold (say 15% or 25%) of household's income? Were there any studies that estimated CHE using 40% of the household's capacity to pay approach? How were such studies dealt with?

**Response 8**

We agree with you that there is no consensus regarding the threshold of CHS. We conducted meta-analyses on studies that used the same measurement definition of financial hardship for a specific NTD. This was possible only for studies that quantified financial hardship using the same measurement definition for a particular NTD. For example, we performed a meta-analysis to calculate the pooled prevalence of households experiencing CHS due to visceral leishmaniasis based on two studies that defined CHS as OOP costs exceeding 10% of annual household income. The remaining studies were not meta-analyzed due to the differences in the definitions of CHS. Other thresholds or definitions were reported in only one study, making it impossible to perform a meta-analysis. Moreover, all studies used the budget share approach. So, we could not perform a meta-analysis based on the estimation methods or different thresholds. We have elaborated on this point in our manuscript. Please see the changes to the manuscript below.

| **Original version** | **Revised version** |
| --- | --- |
| Method, Line 183-185  We performed meta-analyses to calculate the pooled proportions of CHE, which were measured using the same definition, e.g., direct OOP costs exceeded 10% of annual household income. | Method, Line 200-206  We performed meta-analyses to calculate the pooled prevalence of households experiencing financial hardship. However, this was possible only for studies that quantified financial hardship using the same measurement definition for a particular NTD. For example, we performed a meta-analysis to calculate the pooled prevalence of households experiencing CHS due to visceral leishmaniasis based on two studies that defined CHS as direct costs exceeding 10% of annual household income.(8, 15) The remaining studies were not meta-analyzed due to the differences in the definitions of CHS. |
| Results, Line 237-241  CHE was variedly defined across studies in terms of types of costs (medical costs, medical and transportation costs, direct OOP costs, indirect costs, or total costs), thresholds (5%, 10%, 15%, 25%, 30%, 40%, or 100%), timeframe (monthly, quarterly, or annual), capacity to pay (income, consumption, national average annual household expenditure, or international poverty line) and perspective (household or individual). | Results, Line 264-269  CHS was variedly defined across studies in terms of types of costs (medical costs, medical and transportation costs, direct costs, indirect costs, or total costs), thresholds (5%, 10%, 15%, 25%, 30%, 40%, or 100%), timeframe (monthly, quarterly, or annual), household resources (income, consumption, expenditure, national average annual household expenditure, or international poverty line) and perspective (household or individual). All studies used the budget share approach to quantify CHS. |

**Comment 9**

Line 230-233. "Health systems provided the diagnosis and treatment of NTDs free of charge(16, 18, 21, 24) or financial assistance to households,(17, 18) while medical treatment costs of NTDs were highly paid OOP in the other health systems,(19, 20, 22, 23, 25) as presented in Table 3." Authors must mention which health systems (e.g., publicly financed) provided free of charge treatment.

**Response 9**

Thank you for pointing this out. We have rewritten our manuscript to provide more details. Please see the changes to the manuscript below.

| **Original version** | **Revised version** |
| --- | --- |
| Results, Line 230-233  Health systems provided the diagnosis and treatment of NTDs free of charge(16, 18, 21, 24) or financial assistance to households,(17, 18) while medical treatment costs of NTDs were highly paid OOP in the other health systems,(19, 20, 22, 23, 25) as presented in Table 3. | Results, Line 251-260  The health insurance systems or special programs covered some of the costs. The costs for diagnosis and treatment of visceral leishmaniasis were provided free of charge to patients under the publicly financed health insurance system in Nepal(8, 25) and Sudan.(15) In Nigeria, international development partners funded a special program that provided free diagnosis and treatment of Buruli ulcers, as well as accommodation, school funding, and basic allowance.(20) Additionally, the Indian government had a special program that provides financial assistance to families of patients affected by leprosy.(19) However, patients in India had to pay high OOP costs for medical services of leprosy,(19, 23) chikungunya,(21, 26) and lymphatic filariasis.(24) Similarly, patients in Vietnam also paid high OOP costs for the medical treatment of dengue.(22) For more details, refer to Table 3. |

**Comment 10**

The discussion section needs to be elaborated to highlight important policy implications and recommendations.

**Response 10**

We have rewritten our manuscript to highlight important policy implications and recommendations. Please see the changes to the manuscript below.

| **Original version** | **Revised version** |
| --- | --- |
| Discussion, Line 303-309  NTDs placed households at risk of financial catastrophes due to OOP costs. Our systematic review summarized the financial catastrophes from NTDs. Financial catastrophes from NTDs were not comprehensively evaluated as we found that there were only ten studies quantifying the financial catastrophes among households suffering from six out of 20 WHO’s NTDs. Our research supports the need for an economic framework to guide NTD investments.(26) The ability to prioritize investments, informed partially by economic parameters, may appeal to a broad set of stakeholders and help facilitate the process of building coalitions. | Discussion, Line 331-357  NTDs primarily impact populations with limited financial means, yet the literature addressing the financial hardship caused by NTDs is relatively scarce. Our systematic review revealed that there were only ten studies covering six NTDs. We discovered that many households are facing financial hardship as a result of NTDs, despite having access to publicly funded healthcare systems or special NTD programs. The costs related to NTDs resulted in significant financial hardship for these households, mainly due to the high OOP costs associated with the medical treatment. Even in situations where drugs used to treat NTDs were provided free of charge, the costs for supportive care, medical procedures, transportation, and food were still high and could have a devastating financial impact on these households. Moreover, these financial hardship indicators might not fully reflect the financial risk of the population affected by NTDs because many live in poverty or even extreme poverty. Victims of NTDs are usually those who are socially disadvantaged. They need to make trade-offs between suffering from the disease and seeking healthcare because not all victims can afford the costs of NTDs, especially OOP costs for medical treatment and transportation, which could lead to the abandonment of healthcare.(1-3)  The research findings have shown that merely providing funding for treatments of NTDs is insufficient for protecting those affected by NTDs from financial hardship. Therefore, it is crucial to strengthen the entire healthcare system to effectively address the challenges of NTDs and provide financial protection to the victims. Additionally, it is important to encourage and engage communities to change the behavior of those affected by NTDs so that they seek medical assistance at appropriate healthcare facilities instead of relying on traditional healers or not seeking care at all. Our research also supports the need for an economic framework to guide NTD investments.(27) The ability to prioritize investments, informed partially by economic parameters, may appeal to a broad set of stakeholders and help facilitate the process of building coalitions to achieve the WHO’s goal that 90% of the at-risk population is protected against financial hardship caused by NTDs.(1) |

**Response to Reviewer 2 Comments**

This paper describes a systematic review of the Financial Catastrophe among Patients Suffering from Neglected Tropical Diseases. Overall, I found that the paper is clearly written and well conducted study. I have the following comments/suggestions.

**Response**

We would like to thank you for your valuable comments and suggestions. We have revised our manuscript and responded point-by-point to each issue you raised.

**Comment 1**

In my experience, CHE calculations should traditionally use direct costs within the OPP payment component. I have seen papers also include indirect costs within the OPP payment but personally I’m not sure that this is consistent with the foundation of CHE calculations. In the TB literature, they refer to estimates that include indirect costs as catastrophic cost rather than CHE. Assuming I’m not wrong, I think this would be helpful to explore this inconsistency a little more in the discussion.

**Response 1**

We also rewrote our manuscript to address the point of costs captured in the CHE calculation. Please see the changes to the manuscript below.

| **Original version** | **Revised version** |
| --- | --- |
| - | Methods Line 178-186  The financial risk protection metric is intended to capture only the OOP costs for medical services (e.g., treatment and diagnosis costs). However, some studies considered certain types of direct non-medical costs (e.g., transportation, food, and accommodation costs) and indirect costs (e.g., productivity and income losses) when quantifying financial hardship. Some studies also included informal care costs, such as traditional medicine, as OOP costs.(6) Thus, our systematic review categorized costs extracted from the eligible studies as direct costs (OOP costs) and indirect costs. Direct costs were further categorized as direct medical costs and direct non-medical costs. The combination of direct costs and indirect costs was categorized as total costs. |
| Results Line 241-244  The most commonly used definitions of CHE due to NTD were direct OOP costs of a disease episode exceeding 10% of annual household income (three studies)(8, 15, 20) and total costs of a disease episode exceeding 10% of annual household income (three studies).(8, 15, 25)(8, 22) | Results Line 269-272  The most commonly used definitions of CHS caused by NTDs were direct costs exceeding 10% of annual household income (3 studies)(8, 15, 20) and total costs of a disease episode exceeding 10% of annual household income (3 studies).(8, 15, 25) CHS that included only the direct medical costs was reported in two studies.(8, 22) |
| Discussion Line 311-312  CHE is a well-established indicator for financial risk protection of the healthcare system, which was an essential dimension of the UHC. | Discussion Line 359-372  Although there is no consensus regarding the estimation approach and thresholds in quantifying CHS, it is important to note that these differences can significantly impact the findings and consequently impact the applications and implications of the findings.(6, 28) We found that CHS was variedly defined across studies in terms of estimation approach, types of costs, thresholds, timeframe, household resources, and perspective. Our review revealed that 90% of the included studies captured direct non-medical costs as part of the OOP costs. (8, 15, 19-21, 23-26) Furthermore, Seventy percent of the included studies considered indirect costs in quantifying financial hardship.(8, 15, 19, 21, 23, 25, 26) This approach aligned with an indicator called “catastrophic costs” that has emerged in tuberculosis studies. Catastrophic costs occur when the total healthcare costs, including direct and indirect costs, exceed 20% of the annual household income.(28) This indicator could be a more comprehensive measure of the overall financial burden of NTDs on the household beyond just the OOP costs which will be useful when evaluating and monitoring different healthcare policies and interventions to mitigate financial hardship caused by NTDs. |

**Comment 2**

There are two main approaches to CHE calculations. Budget share vs capacity to pay. The results could include which one was used and the differences discussed

**Response 2**

We agree with your suggestion. We have revised our manuscript to present that all studies used the budget share method to quantify CHS and a discussion on the estimation approach. Please see the changes to the manuscript below.

| **Original version** | **Revised version** |
| --- | --- |
| Results, Line 237-241  CHE was variedly defined across studies in terms of types of costs (medical costs, medical and transportation costs, direct OOP costs, indirect costs, or total costs), thresholds (5%, 10%, 15%, 25%, 30%, 40%, or 100%), timeframe (monthly, quarterly, or annual), capacity to pay (income, consumption, national average annual household expenditure, or international poverty line) and perspective (household or individual). | Results, Line 264-269  CHS was variedly defined across studies in terms of types of costs (medical costs, medical and transportation costs, direct costs, indirect costs, or total costs), thresholds (5%, 10%, 15%, 25%, 30%, 40%, or 100%), timeframe (monthly, quarterly, or annual), household resources (income, consumption, expenditure, national average annual household expenditure, or international poverty line) and perspective (household or individual). All studies used the budget share approach to quantify CHS. |
| Discussion Line 311-312  CHE is a well-established indicator for financial risk protection of the healthcare system, which was an essential dimension of the UHC. | Discussion Line 359-372  Although there is no consensus regarding the estimation approach and thresholds in quantifying CHS, it is important to note that these differences can significantly impact the findings and consequently impact the applications and implications of the findings.(6, 28) We found that CHS was variedly defined across studies in terms of estimation approach, types of costs, thresholds, timeframe, household resources, and perspective. Our review revealed that 90% of the included studies captured direct non-medical costs as part of the OOP costs. (8, 15, 19-21, 23-26) Furthermore, Seventy percent of the included studies considered indirect costs in quantifying financial hardship.(8, 15, 19, 21, 23, 25, 26) This approach aligned with an indicator called “catastrophic costs” that has emerged in tuberculosis studies. Catastrophic costs occur when the total healthcare costs, including direct and indirect costs, exceed 20% of the annual household income.(28) This indicator could be a more comprehensive measure of the overall financial burden of NTDs on the household beyond just the OOP costs which will be useful when evaluating and monitoring different healthcare policies and interventions to mitigate financial hardship caused by NTDs. |

**Comment 3**

I would consider adding Meta-analysis into the title

**Response 3**

We agree with your suggestion. We have revised the title accordingly.

| **Original version** | **Revised version** |
| --- | --- |
| Title  Financial Catastrophe among Patients Suffering from Neglected Tropical Diseases: A Systematic Review of Global Literature | Title  Financial Hardship among Patients Suffering from Neglected Tropical Diseases: A Systematic Review and Meta-analysis of Global Literature |

**Comment 4**

I would say that dengue is the NTD and dengue shock is one of its conditions. Please check this and if necessary change dengue shock to dengue.

**Response 4**

We agree with your suggestion. We have revised the term “dengue shock” to “dengue” throughout the manuscript.

**Comment 5**

ABSTRACT: RESULTS: “Costs of VL impoverished approximately one-fifth of households”. Please rephrase

**Response 5**

We have revised our abstract and author summary accordingly. Please see the changes to the abstract below.

| **Original version** | **Revised version** |
| --- | --- |
| Abstract, Line 51-52  Costs of VL impoverished approximately one-fifth of households. | Abstract, Line 52-53  Costs of visceral leishmaniasis impoverished 20-26% of the 61 households investigated, depending on the costs captured. |
| Author summary, Line 68-69  Costs of VL impoverished approximately one-fifth of households. | Author summary, Line 68-69  Costs of visceral leishmaniasis impoverished 20-26% of the 61 households investigated, depending on the costs captured. |
| Results, Line 279-281  Costs of visceral leishmaniasis impoverished approximately one-fifth of households depending on the OOP costs captured (20% medical costs, 21% medical and transportation costs, 26% direct OOP costs), as shown in Table 2. | Results, Line 309-311  Costs of visceral leishmaniasis impoverished 20-26% of the 61 households investigated, depending on the costs captured (20% medical costs, 21% medical and transportation costs, 26% direct costs), as shown in Table 2. |

**Comment 6**

MINOR

“ABSTRACT AND AUTHOR SUMMARY: Introduction: Neglected tropical diseases (NTDs) mainly affect underprivileged populations, resulting in catastrophic health expenditure (CHE) and impoverishment from out-of-pocket (OOP) costs. This systematic review aimed to summarize the financial catastrophes from NTDs.” Not all NTDs lead to CHE. I would consider changing this to “potentially resulting in catastrophic health expenditure (CHE) and impoverishment from out-of-pocket (OOP) costs”

**Response 6**

We have revised our abstract and author summary accordingly. Please see the changes to the abstract below.

| **Original version** | **Revised version** |
| --- | --- |
| Abstract, Line 36-38  Neglected tropical diseases (NTDs) mainly affect underprivileged populations, resulting in catastrophic health expenditure (CHE) and impoverishment from out-of-pocket (OOP) costs. | Abstract, Line 35-37  Neglected tropical diseases (NTDs) mainly affect underprivileged populations, potentially resulting in catastrophic health spending (CHS) and impoverishment from out-of-pocket (OOP) costs. |
| Author summary, Line 61-62  Neglected tropical diseases (NTDs) mainly affect underprivileged populations, resulting in catastrophic health expenditure (CHE) and impoverishment from out-of-pocket (OOP) costs. | Author summary, Line 61-63  Neglected tropical diseases (NTDs) mainly affect underprivileged populations, potentially resulting in catastrophic health spending (CHS) and impoverishment from out-of-pocket (OOP) costs. |

**Comment 7**

INTRO: “For example, as in the study reported before the first road map was published, every low-income country was affected by at least five NTDs.” – slightly unclear, please rephrase. **Response 7**

We have rewritten our manuscript accordingly. Please see the changes to the manuscript below.

| **Original version** | **Revised version** |
| --- | --- |
| Introduction, Line 80-82  For example, as in the study reported before the first road map was published, every low-income country was affected by at least five NTDs.(2) | Introduction, Line 80-81  It has been reported that every low-income country is affected by at least five NTDs |

**Comment 8**

In my opinion, the finding that “Financial catastrophes from NTDs were not comprehensively evaluated” should be a clear conclusion within the abstract/author summary (if this is possible with the word limit).

**Response 8**

We have revised our abstract and author summary accordingly. Please see the changes to the abstract below.

| **Original version** | **Revised version** |
| --- | --- |
| Abstract, Line 54-57  **Conclusion:** NTDs lead to a substantial number of households facing financial catastrophes. To develop evidence-informed strategies to minimize the financial catastrophes from NTDs, studies should evaluate the factors contributing to financial catastrophes across household characteristics, disease stages, and treatment-seeking behaviors. | Abstract, Line 55-59  **Conclusion:** NTDs lead to a substantial number of households facing financial hardship. However, financial hardship caused by NTDs was not comprehensively evaluated in the literature. To develop evidence-informed strategies to minimize the financial hardship from NTDs, studies should evaluate the factors contributing to financial hardship across household characteristics, disease stages, and treatment-seeking behaviors. |
| Author summary, Line 69-72  Therefore, to develop evidence-informed strategies to minimize the financial catastrophes from NTDs, studies should evaluate the factors contributing to financial catastrophes across household characteristics, disease stages, and treatment-seeking behaviors. | Author summary, Line 69-73  Nevertheless, financial hardship caused by NTDs were not comprehensively evaluated in the literature. Therefore, to develop evidence-informed strategies to minimize the financial hardship from NTDs, studies should evaluate the factors contributing to financial hardship across household characteristics, disease stages, and treatment-seeking behaviors. |
| Conclusion, Line 349-351  NTDs can be a devastating burden on households, not only in terms of physical and mental health but also financially. NTDs lead to a substantial number of households facing financial catastrophes. | Conclusion, Line 396-399  NTDs can be a devastating burden on households, not only in terms of physical and mental health but also financially. NTDs lead to a substantial number of households facing financial hardship. However, financial hardship caused by NTDs was not comprehensively evaluated in the literature. |
